# Platelet index ratios in HIV: Emerging biomarkers for immune health and disease management

**DOI:** 10.1097/MD.0000000000037576

**Published:** 2024-03-22

**Authors:** Emmanuel Ifeanyi Obeagu, Getrude Uzoma Obeagu

**Affiliations:** a Department of Medical Laboratory Science, Kampala International University, Kampala, Uganda; b School of Nursing Science, Kampala International University, Kampala, Uganda.

**Keywords:** biomarkers, disease management, health, HIV, immune, platelet index ratios

## Abstract

Human Immunodeficiency Virus (HIV) infection is a global health challenge that requires continuous advancements in diagnostic and prognostic tools. Traditional markers, such as CD4 cell counts and viral load, have played a crucial role in monitoring disease progression and guiding therapeutic interventions. However, emerging research suggests that platelet index ratios may serve as valuable biomarkers in assessing immune health and managing HIV-associated complications. This paper explores the significance of platelet index ratios, including platelet-to-lymphocyte ratio and mean platelet volume-to-lymphocyte ratio, as potential indicators of immune system status in individuals living with HIV. The interplay between platelets, lymphocytes, and their ratios reflects the dynamic nature of the immune response and inflammatory processes during HIV infection. Understanding the role of platelet index ratios in HIV could lead to the development of accessible and cost-effective biomarkers for monitoring immune health. Implementation of these ratios in routine clinical practice may enhance the precision of disease prognosis and guide personalized treatment strategies. Additionally, the exploration of platelet index ratios may pave the way for innovative therapeutic interventions aimed at modulating immune responses in HIV-infected individuals. In conclusion, platelet index ratios represent promising emerging biomarkers for evaluating immune health and managing HIV-related complications. Further research and clinical validation are warranted to establish the utility of these ratios in routine HIV care, potentially revolutionizing the approach to monitoring and improving the health outcomes of individuals living with HIV.

## 1. Introduction

Human Immunodeficiency Virus (HIV) remains a global health challenge, necessitating continuous innovation in the assessment of immune health, monitoring of disease progression, and guidance of therapeutic interventions. Amidst the complex landscape of HIV care, emerging biomarkers offer promising avenues for improved patient management. Platelet index ratios, including platelet-to-lymphocyte ratio (PLR) and mean platelet volume-to-platelet count ratio (MPV/PC), have emerged as potential game-changers in the field of HIV research and treatment.^[1–5]^ Traditionally associated with hemostasis and thrombosis, platelet index ratios have recently transcended their classical roles. These ratios are increasingly recognized for their ability to reflect the interplay between platelets, lymphocytes, and overall inflammatory processes within the body. Understanding their clinical significance and applications in the context of HIV is paramount.^[6]^

This paper aims to provide a thorough exploration of the utilization of platelet index ratios as emerging biomarkers in HIV care. By delving into their implications for immune health assessment, early disease progression monitoring, treatment decision-making, prognostic value, and their role in personalized medicine, we aim to shed light on their potential to revolutionize HIV management. Furthermore, as ongoing research continues to unveil their evolving roles, this publication will discuss the future directions of platelet index ratios in HIV care, emphasizing the need for multidisciplinary research and clinical applications. As the world collectively strives to enhance the quality of life and health outcomes for those living with HIV, the exploration of platelet index ratios offers a promising avenue for refining disease management and personalized treatment strategies.^[7]^ By illuminating the multifaceted and evolving roles of these emerging biomarkers, this publication aims to contribute to ongoing endeavors to optimize HIV care, monitor disease progression, and ultimately improve the lives of those affected by this persistent global health challenge.

## 2. Aim

The aim of this review is to comprehensively assess the potential of platelet index ratios, specifically the PLR and mean platelet volume-to-lymphocyte ratio, as emerging biomarkers for evaluating immune health and aiding in disease management among individuals infected with HIV. The primary objectives include:

### 2.1. Platelets in HIV pathogenesis

Platelets, traditionally recognized for their role in hemostasis and blood clotting, have emerged as essential players in the pathogenesis of HIV infection. While HIV primarily targets and infects CD4+ T cells, the virus also influences platelet function and activation. Understanding the interplay between HIV and platelets is crucial for a comprehensive grasp of the disease’s pathogenesis.^[8–10]^ Platelets have immune-regulatory functions, including the ability to interact with various immune cells, such as monocytes, neutrophils, and lymphocytes. This interaction can modulate the immune response to HIV.^[11]^ HIV infection triggers platelet activation and the release of platelet-derived factors, such as platelet factor 4 (PF4) and β-thromboglobulin. This activation may contribute to the systemic inflammation and immune activation seen in HIV-infected individuals.^[12]^ Recent study has shown that platelets express CD4 receptors and co-receptors, which are required for HIV attachment and internalization. While platelets are not a primary target for HIV infection, viral particles can interact with and be internalized by platelets.^[13]^ Thrombocytopenia (a low platelet count [PC]) is a common hematological complication in HIV infection. It can result from both immune-mediated platelet destruction and decreased platelet production in the bone marrow. The immune-mediated destruction may be due to antiplatelet antibodies.^[14]^

Platelets can release pro-inflammatory molecules, such as chemokines and cytokines, which contribute to the chronic inflammation observed in HIV-infected individuals. This chronic inflammation is associated with a range of complications and comorbidities.^[15]^ Individuals with HIV are at an increased risk of coagulation abnormalities and thrombotic events, possibly due to platelet activation, inflammation, and the presence of procoagulant microparticles in circulation.^[16]^ There is evidence that platelets can carry HIV virions and transport them to various tissues and organs. This transport mechanism may play a role in viral dissemination.^[17,18]^

### 2.2. The specific correlation between platelets and immune function in HIV

The correlation between platelets and immune function in HIV is a complex interplay that reflects the intricate dynamics of the immune system during the course of infection. Thrombocytopenia, a condition characterized by low PCs, is common in individuals with HIV. Thrombocytopenia is often associated with advanced stages of HIV/AIDS, suggesting a correlation between low PCs and immune system suppression. HIV infection induces systemic immune activation. Activated immune cells release pro-inflammatory cytokines and other mediators that can activate platelets. Elevated levels of platelet activation markers, such as P-selectin and CD40L, have been observed in HIV-infected individuals, indicating a potential link between immune activation and platelet function. Platelets play a role in the inflammatory response by interacting with immune cells and releasing cytokines. HIV-related inflammation may contribute to a prothrombotic state, leading to increased platelet activation and potential involvement in coagulation abnormalities.^[15,16]^

Platelets can interact with various immune cells, including T cells and monocytes, modulating their function. Platelets may contribute to the regulation of immune responses, influencing cytokine production and immune cell activation. PLR, a platelet index ratio, has been proposed as a potential biomarker for immune health in HIV. Studies suggest that an elevated PLR may correlate with lower CD4 counts, indicating a potential association between platelet indices and immune status. The correlation between platelets and immune function may have predictive value for disease progression and the risk of opportunistic infections. Changes in platelet indices might be indicative of treatment response or failure, providing additional insights into the effectiveness of antiretroviral therapy (ART).^[17,18]^

### 2.3. Advantages of platelets indicators related to HIV compared to other immune indicators such as current CD4 T cell counts

Platelet indicators, specifically platelet indices such as PLR and mean platelet volume-to-lymphocyte ratio, offer several potential advantages compared to traditional immune indicators, such as CD4 T cell counts, in the context of HIV. Platelet indices may exhibit dynamic changes in response to immune alterations, potentially allowing for early detection of immune dysfunction before significant changes occur in CD4 T cell counts. Platelets actively participate in the inflammatory response, and platelet indices reflect the ongoing inflammation associated with HIV. This provides a broader perspective on the immune system’s status beyond the specific measurement of T cell counts. Platelet activation markers can serve as indicators of overall immune activation. This holistic assessment goes beyond the isolated measurement of CD4 T cell counts, providing a more comprehensive view of immune system activity. Platelet indices have shown potential as predictive markers for the risk of opportunistic infections in HIV-infected individuals. This predictive value may offer insights into the overall immune competence of individuals, complementing CD4 T cell counts. Platelet indices may provide insights into non-AIDS-related complications and comorbidities associated with HIV, contributing to a more holistic understanding of the impact of HIV on the immune system and overall health.^[15–17]^

Platelet indices can be derived from routine complete blood count tests, which are widely available and more cost-effective than specialized tests for measuring CD4 T cell counts. This accessibility facilitates regular monitoring of immune health. Changes in platelet indices may dynamically respond to ART, offering an additional parameter for monitoring treatment response beyond CD4 T cell counts. This can contribute to a more nuanced evaluation of the effectiveness of therapeutic interventions. Platelet indices may provide individualized insights into immune responses, allowing for a more personalized approach to HIV management based on the unique characteristics of a patient’s immune system.^[18]^

### 2.4. Platelet index ratios in HIV care

Platelet index ratios, such as PLR and MPV/PC, have gained significance in the care and management of HIV infection.^[19]^ PLR and MPV/PC ratios provide a snapshot of the balance between platelets and lymphocytes, as well as the size of platelets in the bloodstream. These ratios can offer valuable information about the immune status of HIV-infected individuals. A low PLR and an elevated MPV/PC may indicate immune dysfunction and ongoing inflammation.^[20]^ Changes in platelet index ratios over time can serve as early indicators of disease progression. In HIV, deviations from baseline values can signal more advanced disease stages. Monitoring these changes can prompt healthcare providers to consider adjustments to ART or other treatment modalities.^[21]^ Platelet index ratios can guide treatment strategies. A lower PLR and an elevated MPV/PC may influence the decision to initiate or modify ART. Understanding the impact of ART on these ratios is crucial for optimizing patient care.^[22]^

As effective ART often leads to immune recovery, monitoring changes in platelet index ratios can provide insights into the response to treatment. An increasing PLR and normalization of MPV/PC may signify improved immune health.^[23]^ Platelet index ratios have been linked to various HIV-related complications, such as cardiovascular disease and neurocognitive impairments. Elevated PLR and altered MPV/PC may indicate an increased risk of these complications, providing opportunities for early intervention.^[24]^ Understanding platelet index ratios in the context of HIV can contribute to personalized treatment strategies. Tailoring treatment based on an individual’s unique platelet profile and other clinical parameters can optimize therapeutic outcomes.^[25,26]^ Platelet index ratios, such as PLR and MPV/PC, offer valuable insights into immune health and disease progression in individuals living with HIV. As our understanding of these ratios continues to evolve, they have the potential to enhance the quality of care and contribute to more personalized treatment strategies for those affected by HIV (Table 1).^[27]^

**Table 1:** Platelet index and platelet-to-lymphocytes ratio (PLR) in HIV.^[42]^

CD4 count (cells/mm³)	Viral load (copies/mL)	Platelet count (×10^9^/L)	Lymphocyte count (cells/mm³)	Platelet-to-lymphocyte ratio (PLR)	Mean platelet volume (MPV)	Platelet distribution width (PDW)
350	120,000	180	800	0.225	10.2	14.5
500	80,000	250	900	0.278	9.8	12.3
200	150,000	120	600	0.200	11.5	13.0
450	40,000	200	850	0.235	10.0	15.2
300	200,000	150	700	0.214	10.8	14.0

### 2.5. PLR in HIV

The PLR is an emerging biomarker that has gained attention in the context of HIV infection. PLR is a simple yet informative index that reflects the balance between PCs and lymphocyte counts in the bloodstream. While traditionally used as a marker of inflammation and immune response in other medical conditions, PLR has shown potential implications in HIV care and research^[28]^ PLR can provide valuable insights into the overall immune health of individuals living with HIV. It is a reflection of the immune status beyond traditional CD4 cell counts and viral load measurements. A higher PLR may suggest an imbalance in the immune response, indicating the presence of systemic inflammation or immune activation, both of which are common features in HIV infection.^[29]^ Changes in PLR over time may serve as early indicators of disease progression in HIV. Deviations from baseline values can signal the advancement of HIV infection. Monitoring PLR alongside other clinical markers can prompt healthcare providers to consider initiating or modifying ART when needed.^[30]^

PLR has the potential to guide treatment decisions. A higher PLR may influence the decision to initiate or modify ART. Understanding how ART impacts PLR can help optimize patient care and tailor treatment regimens.^[31]^ Ongoing research explores the use of PLR as a prognostic marker in HIV. High PLR values may be associated with a greater risk of HIV-related complications and non-AIDS conditions, making it a valuable tool for predicting long-term outcomes.^[32,33]^ PLR is a promising biomarker with potential applications in assessing immune health, monitoring disease progression, and guiding treatment decisions in HIV.^[34]^ As research in this field continues to evolve, PLR has the potential to enhance the quality of HIV care and contribute to more personalized treatment strategies for individuals living with HIV.

### 2.6. MPV/PC in HIV

The MPV/PC is an emerging biomarker that has shown potential relevance in the context of HIV infection. MPV represents the average size of platelets in the bloodstream, and when evaluated in conjunction with PC, it can offer insights into various aspects of HIV care and research:^[35]^ MPV/PC ratio can serve as an indicator of the inflammatory and immune response in HIV-infected individuals. A higher MPV/PC ratio may suggest platelet activation and changes in platelet size, reflecting ongoing immune system activity and inflammation.^[36]^ Changes in MPV/PC ratios over time may provide valuable information about disease progression in HIV. Deviations from baseline values can signal more advanced disease stages and the presence of inflammatory processes.^[37]^ Monitoring MPV/PC ratios during ART can help assess the response to treatment. An increasing MPV/PC ratio may indicate positive immunological changes and the effectiveness of antiretroviral therapy.^[38]^

Elevated MPV/PC ratios may be associated with an increased risk of complications and comorbidities commonly observed in HIV, such as cardiovascular disease. Monitoring MPV/PC ratios can help identify individuals at a higher risk of developing non-AIDS-related conditions.^[39]^ The MPV/PC ratio holds potential for early intervention strategies. When used in combination with other clinical markers, it can provide timely indications of disease progression and complications, allowing healthcare providers to make informed decisions about patient management.^[40]^ Future research in this field is likely to focus on refining the clinical utility of MPV/PC ratios in HIV care, as well as their role in predicting long-term outcomes and guiding personalized treatment strategies.^[37]^ The MPV/PC ratio is an emerging biomarker with potential applications in assessing immune health, monitoring disease progression, and guiding treatment decisions in HIV. As research in this field continues to evolve, the MPV/PC ratio has the potential to enhance the quality of care and contribute to more personalized treatment strategies for individuals living with HIV.^[41]^

### 2.7. Platelets ratios as emerging biomarkers for HIV

Platelet index ratios, such as PLR and MPV/PC, are emerging as potential biomarkers in the context of HIV. These ratios, traditionally associated with hemostasis and thrombosis, are now recognized for their broader significance in HIV as emerging biomarkers with several implications:^[42]^ PLR and MPV/PC ratios offer insights into the balance between platelets and lymphocytes, as well as platelet size. These ratios provide a new perspective on immune health, as deviations from normal values can signify immune dysfunction and ongoing inflammation. Changes in platelet index ratios may serve as early indicators of disease progression. Altered ratios can signal more advanced stages of HIV infection, prompting clinicians to consider initiating or modifying ART. Platelet index ratios can guide treatment decisions, potentially influencing the initiation or modification of ART. Understanding how ART impacts these ratios is critical for optimizing patient care.

Effective ART often leads to an improvement in platelet index ratios, signifying immune recovery. Monitoring these changes can provide valuable insights into the response to treatment and overall immunological health. These emerging ratios have been associated with various HIV-related complications, including cardiovascular disease, neurocognitive impairments, and non-AIDS-related malignancies. Elevated PLR and altered MPV/PC may indicate an increased risk of these complications, allowing for early intervention. Recognizing the role of platelet index ratios in HIV care contributes to personalized treatment strategies. Tailoring treatment based on an individual’s unique platelet profile and other clinical parameters can optimize therapeutic outcomes. As the understanding of platelet index ratios in HIV evolves, ongoing research explores their potential as prognostic markers, early indicators of complications, and tools for personalized treatment. Future research is expected to refine their clinical utility and expand their role in HIV care.^[27]^

### 2.8. Other infectious pathology in humans in which platelet index ratios are markers

Platelet index ratios can serve as markers not only in HIV but also in various other infectious pathologies.^[43]^ These ratios, including PLR, mean platelet volume (MPV), and platelet distribution width (PDW), may be indicative of inflammatory and infectious conditions. Elevated PLR and alterations in MPV and PDW have been associated with sepsis, indicating the presence of a systemic bacterial infection.^[44]^ Altered platelet indices may be observed in viral hepatitis infections, with decreased PCs and changes in MPV.^[45]^ Thrombocytopenia (reduced PC) is a common feature in dengue virus infections, affecting PLR and other platelet indices. Thrombocytopenia and changes in platelet indices are common in malaria infections, reflecting the impact of the parasite on the hematological system. Elevated PLR and alterations in MPV have been observed in severe cases of COVID-19, serving as potential markers of disease severity.^[46]^ Certain fungal infections, especially those causing systemic mycoses, may impact platelet indices, contributing to thrombocytopenia and altered PLR. Patients with inflammatory bowel diseases may experience changes in platelet indices, reflecting the inflammatory nature of these conditions. Platelet indices may be influenced by severe urinary tract infections, with alterations in PLR and MPV observed in some cases.^[47]^ Patients with persistent fever of unknown origin may exhibit changes in platelet indices, which can be suggestive of an underlying infectious pathology.

### 2.9. Other infectious pathology in humans in which platelet index ratios are markers

Platelet index ratios can serve as markers not only in HIV but also in various other infectious pathologies.^[43]^ These ratios, including PLR, MPV, and PDW, may be indicative of inflammatory and infectious conditions. Elevated PLR and alterations in MPV and PDW have been associated with sepsis, indicating the presence of a systemic bacterial infection.^[44]^ Altered platelet indices may be observed in viral hepatitis infections, with decreased PCs and changes in MPV.^[45]^ Thrombocytopenia (reduced PC) is a common feature in dengue virus infections, affecting PLR and other platelet indices. Thrombocytopenia and changes in platelet indices are common in malaria infections, reflecting the impact of the parasite on the hematological system. Elevated PLR and alterations in MPV have been observed in severe cases of COVID-19, serving as potential markers of disease severity.^[46]^ Certain fungal infections, especially those causing systemic mycoses, may impact platelet indices, contributing to thrombocytopenia and altered PLR. Patients with inflammatory bowel diseases may experience changes in platelet indices, reflecting the inflammatory nature of these conditions. Platelet indices may be influenced by severe urinary tract infections, with alterations in PLR and MPV observed in some cases. Patients with persistent fever of unknown origin may exhibit changes in platelet indices, which can be suggestive of an underlying infectious pathology.

### 2.10. Platelet index ratios and future research directions

The exploration of platelet index ratios in the context of HIV is a dynamic field with promising potential for future research directions. As our understanding of these ratios continues to evolve, several important areas of research and development are likely to shape the future of their utility in HIV care.^[48]^ Conducting large-scale, long-term studies that track platelet index ratios over extended periods in diverse HIV populations can help further our understanding of their clinical significance. These studies can provide insights into how these ratios change over time and whether they correlate with clinical outcomes, such as disease progression and the development of complications. Research can delve into the identification of specific clinical scenarios where platelet index ratios offer the most value. Understanding which patient groups or disease stages benefit most from the use of these ratios can guide their clinical application and provide more targeted insights. Investigating the interplay between platelet index ratios and other immunological and hematological biomarkers is essential. Comprehending how these ratios relate to markers such as CD4 counts, viral load, and inflammatory markers can provide a more comprehensive picture of immune health and disease progression.

Future research should focus on understanding how different ART regimens impact platelet index ratios. This can help optimize treatment choices and assess which regimens are associated with the most favorable changes in these ratios, signifying improved immune health. Ongoing research into the potential of platelet index ratios as prognostic markers can aid in predicting disease outcomes and complications. Developing robust prognostic models that incorporate these ratios could provide clinicians with valuable tools for patient management and early intervention. Investigating the use of platelet index ratios as part of a personalized medicine approach for HIV care is a promising avenue. Research can explore how to tailor treatment strategies based on an individual’s unique platelet profile and other clinical parameters.^[49]^

## 3. Conclusion

In conclusion, the comprehensive review of platelet index ratios in the context of HIV underscores their evolving role as emerging biomarkers with multifaceted implications for immune health and disease management. The exploration of platelet index ratios, including PLR and MPV/PC, has illuminated the potential for these ratios to revolutionize our approach to HIV care.

## Author contributions

**Conceptualization:** Emmanuel Ifeanyi Obeagu.

**Methodology:** Emmanuel Ifeanyi Obeagu, Getrude Uzoma Obeagu.

**Supervision:** Emmanuel Ifeanyi Obeagu.

**Visualization:** Emmanuel Ifeanyi Obeagu.

**Writing – original draft:** Emmanuel Ifeanyi Obeagu, Getrude Uzoma Obeagu.

**Writing – review & editing:** Emmanuel Ifeanyi Obeagu, Getrude Uzoma Obeagu.
